# Source Identification of *Klebsiella pneumoniae* Causing Six Episodes of Recurrent Sepsis in an Adolescent That Underwent Hematopoietic Stem Cell Transplantation

**DOI:** 10.3390/pathogens10091123

**Published:** 2021-09-02

**Authors:** Suejung Jo, Hyun Mi Kang, Seong Koo Kim, Jae Wook Lee, Nack-Gyun Chung, Bin Cho, Dae Chul Jeong, Yeon-Joon Park

**Affiliations:** 1 Department of Pediatrics, College of Medicine, The Catholic University of Korea, Seoul 06591, Korea; skdmlwls1@naver.com (S.J.); ksk3497@catholic.ac.kr (S.K.K.); dashwood@catholic.ac.kr (J.W.L.); cngped@catholic.ac.kr (N.-G.C.); chobinkr@catholic.ac.kr (B.C.); dcjeong@catholic.ac.kr (D.C.J.); 2 Department of Laboratory Medicine, College of Medicine, The Catholic University of Korea, Seoul 06591, Korea; yjpk@catholic.ac.kr

**Keywords:** child, immunocompromised, *Klebsiella pneumoniae*, sepsis

## Abstract

Septicemia or bacteremia is one of the leading causes of death worldwide. Long-term tunneled central venous catheters (CVCs) are usually placed in children undergoing chemotherapy or hematopoietic stem cell transplantation (HSCT) for underlying hemato–oncologic malignancies. However, catheter-related complications have been reported frequently, and there is high morbidity and mortality related to catheter-line-associated bloodstream infections (CLABSIs). We report a rare case of six episodes of recurrent *K. pneumoniae* sepsis within a 6-month period in a 12-year-old male adolescent that underwent HSCT for acute lymphoblastic leukemia, despite treatment with susceptible antibiotics. The patient received extensive diagnostic evaluations to find the hidden source; however, failure to discover the primary source led to multiple recurrences. Through enterobacterial repetitive intergenic consensus (ERIC)-PCR, we were able to identify the relationship between the six episodes and recognize the source of bacteremia.

## 1. Introduction

Septicemia or bacteremia is one of the leading causes of death worldwide, and 6–10% of the patients who experience one episode of Gram-negative bacteremia will have another recurrent episode [1]. There are two factors to consider in recurrent bacteremia—the pathogen factor and the host factor. Recurrent bacteremia by the same pathogen may be due to ineffective therapy caused by the failure to eliminate the source of infection due to insufficient duration of therapy, antibiotic choice, or inducible antibiotic resistance by the pathogen. The host’s immunity status and the presence of indwelling catheters may also be important risk factors for recurrent bacteremia [2,3].

Long-term tunneled central venous catheters (CVCs) are usually placed in children undergoing chemotherapy or hematopoietic stem cell transplantation (HSCT) for underlying hemato–oncologic malignancies. However, catheter-related complications such as malfunction, thrombosis, and catheter-line-associated bloodstream infections (CLABSIs) have been reported in up to 15% of the patients [4]. Mortality related to CLABSIs has been reported to occur in up to 25% of the cases [5]; therefore, careful surveillance and monitoring of CLABSIs are crucial, especially in immunocompromised or critical patients. 

We report a case of six episodes of recurrent *Klebsiella pneumoniae* sepsis in a patient that underwent HSCT. Through enterobacterial repetitive intergenic consensus (ERIC)-PCR, we were able to identify the relationship between the six episodes and recognize the source of bacteremia. 

## 2. Case

**Figure 1:**
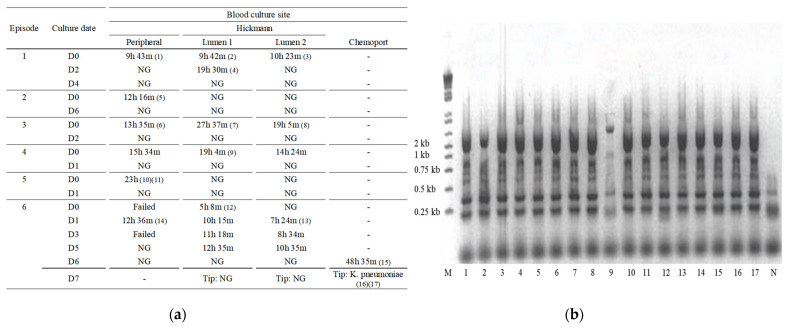
(**a**) Time to bacterial growth at each episode by culture site. In episode 1, one lumen of the Hickman catheter was initially positive; however, the differential time to positivity, compared to the peripheral culture, was <1 h. In episodes 2–5, only the peripheral blood cultures were positive, or the peripheral cultures were positive before central cultures. Thus, time to bacterial growth in episodes 1–5 suggested Hickman catheter-nonrelated bloodstream infections. The numbers within the parentheses correlate with the specimen number of the ERIC-PCR agarose gel electrophoresis image; (**b**) DNA fingerprints of *K. pneumoniae* isolates from the patients, generated by ERIC-PCR. which shows that other than specimen 9, the exact same *K. pneumoniae* clone was the causative pathogen. D, day; h, hours; m, minutes; M, size marker 1 kb ladders; N, negative control; NG, no growth.

A 12-year-old male patient visited the emergency department (ED) with a fever of 39.3 °C. Four months prior, he had undergone unrelated peripheral blood stem cell transplantation (uPBSCT) for acute lymphoblastic leukemia with central nervous system relapse. At the ED, his complete blood cell count showed neutropenia. Blood culture was drawn from his peripheral blood and from both lumens of the indwelling Hickman catheter. At 10 h after the blood cultures were drawn, both the peripheral and Hickman catheter blood cultures showed presumptive bacterial growth, later identified as *K. pneumoniae*. The differential time to positivity of the central and peripheral blood cultures was <1 h suggesting catheter-nonrelated bacteremia. He was administered susceptible intravenous antibiotics (Table 1) for two weeks and successfully treated for the first episode of *K. pneumoniae* sepsis (Figure 1a).

Three weeks after the initial episode, the patient presented to the ED with fever, abdominal pain, diarrhea, and neutropenia. After peripheral and Hickman catheter blood cultures were drawn, he was initiated with piperacillin/tazobactam and amikacin immediately. Within 13 h, his peripheral blood culture showed presumptive bacterial growth, later identified as *K. pneumoniae*. As blood drawn from both lumens of the Hickman catheter were negative, he was treated for catheter-nonrelated bacteremia. The antibiotic susceptibility patterns of the *K. pneumoniae* cultured showed a different profile, compared to the *K. pneumoniae* isolated from the first episode (Table 1). Therefore, he was given an abdominal computed tomography to rule out any deep focal infection source; however, there were no abnormal findings. He was administered intravenous tigecycline for two weeks and successfully treated for his second episode of *K. pneumoniae* sepsis.

Four weeks after the second episode, the patient experienced an acute onset of fever ≥39.5 °C. He immediately returned to the ED and was initiated with antibiotics after blood cultures were drawn. After 13 h, *K. pneumoniae* was cultured from his peripheral blood, and 19 h later, his central blood cultures were positive. He was treated for his third episode of catheter-nonrelated bacteremia.

Exactly 4 weeks after the third episode, the patient revisited the ED for a fever reaching 40 °C. Again, blood culture revealed catheter-nonrelated *K. pneumoniae* bacteremia. After initiating antibiotics for the fourth episode, the patient became afebrile and his general condition improved within 48 h. Immediate negative conversion of his blood cultures was observed at 24 h follow-up. However, within 96 h after negative conversion, his fever spiked to 39.7 °C, and his peripherally drawn blood cultures revealed a fifth episode of catheter-nonrelated *K. pneumoniae* bacteremia.

A full physical examination revealed no rash, no abdominal tenderness, or any signs of focal infections. In order to find the source of *K. pneumoniae* causing recurrent sepsis, he was given echocardiography, revealing no vegetation or any other evidence of infective endocarditis. As he was neutropenic at all five episodes, the source was concluded to be bacterial translocation from his gastrointestinal tract during neutropenia. The patient was administered a prolonged 21-day course of susceptible antibiotics and was discharged.

Seven weeks later, the patient visited the ED with a fever of 39.5 °C and no other symptoms. His CBC revealed no leukopenia or neutropenia, unlike the previous episodes (Table 2). The patient remained febrile for 5 days into the antibiotic treatment and repeated cultures revealed persistent bacteremia. He underwent a follow-up abdominal CT, ocular examination, and echocardiography, which all excluded any possible sources of infection. On the 6th day after fever onset, a full physical examination led to the discovery of a chemoport that had not been used since HSCT but had been undergoing routine function checks at the outpatient clinic every month. A chemoport needle was inserted into the injection port beneath the skin, and blood culture was drawn from the port. By this time, the peripheral blood and Hickman lumen blood cultures were negative for any bacteria. Then, 48 h after blood cultures were drawn from the chemoport, results came back positive for *K. pneumoniae*. Both central catheters were removed, and tip cultures from both central lines revealed that the chemoport tip was colonized with *K. pneumoniae*.

In order to understand the relationship between all six episodes of *K. pneumoniae* sepsis, an ERIC-PCR was performed on all the isolates from the six episodes. The ERIC-PCR bands showed that other than specimen 9 from episode 4, the exact same *K. pneumoniae* clone was the causative pathogen, and thus, the source was *K. pneumoniae* colonized at the tip of the chemoport (Figure 1b).

## 3. Methods

The definition for positive differential time to positivity suggesting CLABSI was the detection of bacterial growth from a blood culture drawn from the CVC 2 h prior to detection of bacterial growth from a blood culture drawn from the peripheral vein [6]. For blood cultures, at least 5–10 mL of blood was drawn from the peripheral vein and CVC of the patient. The culture bottles were then inserted into the incubators at the same time. When the alarm went off for a presumptive bacterial growth in any of the culture bottles, the time was recorded to monitor differential time to positivity. The Vitek-2 (bioMérieux, Marcy l’Etoile, France) was used for species identification and antimicrobial susceptibility tests. The Clinical and Laboratory Standards Institute (CLSI) guideline was used to determine the cutoff for antibiotic susceptibilities and the presence of ESBL-producing *K. pneumoniae* [7]. All *K. pneumoniae* isolates prospectively stored underwent ERIC-PCR using the methods and conditions previously published by Kim et al. [8].

## 4. Discussion

This was a rare case of six episodes of recurrent *K. pneumoniae* sepsis within a 6-month period in a 12-year-old male adolescent that underwent HSCT for acute lymphoblastic leukemia, despite treatment with susceptible antibiotics. The patient received extensive diagnostic evaluations to find the hidden source; however, failure to discover the primary source led to multiple recurrences. The colonization of *K. pneumoniae* on the tip of a hidden chemoport was eventually found to be the source.

Identifying and controlling the source of infection are critical steps in the management of sepsis [9]. However, in immunocompromised patients with neutropenia, especially after HSCT or chemotherapy when the oral and gut mucosa are friable, bacterial translocation into the bloodstream can cause sepsis [10]. In this case, the patient was neutropenic during the first five episodes. As the infection source could not be identified after extensive evaluations, bacterial translocation from the gut mucosa was concluded as a possible source. In fact, the *K. pneumoniae* isolates cultured from one of the lumens of the Hickman catheter were found to be a completely different genotype, possibly from bacterial translocation (Figure 1b). This case highlights the need for source identification and complete eradication in order to control recurrent infections.

The overall survival rate of childhood cancer has made significant progress over the past few decades. However, challenges remain in the management of treatment-related infections, which greatly impact the morbidity and mortality of these patients [1,11,12]. As the majority of cancer patients undergo venous access via insertion of CVCs for treatment and blood sampling, they are placed at an even higher risk of infections. In fact, the overall incidence of CLABSIs in cancer patients is estimated at 0.5–10 per 1000 CVC days, and the related mortality ranges from 12 to 40% [13,14]. Furthermore, the presence of multiple CVCs usually implies that more severe underlying conditions may be present. Multiple CVCs lead to more frequent manipulations and procedures, increasing the overall risk of infections in patients that are already at high risk. In this case, the patient underwent HSCT for relapsed leukemia and was receiving function checks of both the Hickman and chemoport catheters at the outpatient clinic. In retrospect, after each function check, the patient experienced episodes of sepsis. However, failure to obtain blood cultures from the chemoport during fever episodes at the ED led to difficulties in discovering the source of infection. Thus, in patients with multiple CVCs, a systematic approach needs to be taken to ensure that blood cultures from all lumens are taken during febrile episodes. Educating patients and guardians about providing information about the presence of all indwelling CVCs during blood cultures and bringing awareness to the medical staff about the importance of obtaining blood cultures from all indwelling CVCs are necessary.

During the final prolonged episode of K. *pneumoniae* sepsis, a full examination of the patient and retrospective review of the previous blood cultures and culture sites led to the discovery that the patient’s chemoport had not been included in the blood cultures during previous episodes. The blood drawn from the chemoport revealed growth of *K. pneumoniae* 6 days after initiating antibiotics; meanwhile, blood cultured from the peripheral vein and all lumens of the Hickman catheter showed no more bacterial growth. An ERIC-PCR was performed on *K. pneumoniae* isolates cultured from all 6 episodes and the chemoport tip revealing identical clones in all but one isolate during the fourth episode. This proved that the chemoport tip was the source of *K. pneumoniae*, and the search for the source of infection was terminated. After the removal of both CVCs, the patient did not have any more recurrences. As shown from this case, there are many challenges associated with diagnosing CLABSI, and multiple indwelling CVCs may augment the difficulties in identifying CVC-associated infections.

A limitation of this case was that not all *K. pneumoniae* isolated from each of the lumens at the episodes were included in the ERIC-PCR for clonal analyses due to the unavailability of some of the isolates for genetic analyses. However, all the isolates with different antibiotic susceptibilities were included, as well as at least one isolate from each of the episodes suggesting identical clones.

## 5. Conclusions

This case highlights the importance of identifying and eradicating the source of infection, which is key to a successful treatment. Moreover, patients with multiple concurrent central lines may be at a higher risk for CLABSIs; therefore, decisions to insert more than one CVCs should be made with caution. However, when it is deemed critical for the patient to have multiple CVCs, a systematic approach should be made on the maintenance and surveillance of the CVCs, and education on the possible complication risks and importance of obtaining blood cultures from all lumens of the CVCs may aid in preventing recurrent episodes of sepsis.

## Figures and Tables

**Table 1:** Antibiogram of *K. pneumoniae* isolates cultured at each sepsis episode.

	Episode No./Culture Site														
Antibiotic Susceptibility	1			2	3			4			5	6			
	P	H1	H2	P	P	H1	H2	P	H1	H2	P	P	H1	H2	Chemoport
Amikacin	S	S	S	I	S	R	R	S	S	S	S	S	S	S	S
Amoxicilin-clavulanic acid	R	R	R	R	R	R	R	R	R	R	R	R	R	R	R
Ampicillin	R	R	R	R	R	R	R	R	R	R	R	R	R	R	R
Aztreonam	R	R	R	R	R	S	S	R	R	R	R	I	S	S	R
Carbapenemase	N	N	N	N	N	N	N	N	N	N	N	N	N	N	N
Cefazolin	R	R	R	R	R	R	R	R	R	R	R	R	R	R	R
Cefepime	S	S	S	R	S	S	S	S	S	S	S	S	S	S	S
Cefotaxime	R	R	R	R	R	R	R	R	R	R	R	R	R	R	R
Cefoxitin	R	R	R	R	R	R	R	R	R	R	R	R	R	R	R
Ceftazidime	R	R	R	R	R	I	I	R	R	R	R	R	R	R	R
Ciprofloxacin	R	R	R	I	R	R	R	R	R	R	R	R	R	R	R
ESBL	N	N	N	N	N	N	N	N	N	N	N	N	N	N	N
Ertapenem	R	R	R	R	R	S	S	I	S	S	I	S	S	S	S
Gentamicin	R	R	R	R	R	R	R	R	R	R	R	R	R	R	R
Imipenem	S	S	S	R	I	S	S	S	S	S	S	S	S	S	S
Meropenem	S	S	S	I	I	S	S	S	S	S	S	S	S	S	S
Piperacillin/tazobactam	I	I	I	R	R	S	S	R	I	I	R	I	I	I	R
Tigecycline	S	S	S	S	S	S	S	S	S	S	S	S	S	S	S
Trimethoprim/sulfamethoxazole	R	R	R	R	R	R	R	R	R	R	R	R	R	R	R

H1, Hickman lumen 1; H2, Hickman lumen 2; I, intermediate; N, negative; P, peripheral; R, resistant; S, sensitive; with reference to the *K. pneumoniae* isolated at episode 1, antibiotic susceptibility patterns that differed were shaded in gray.

**Table 2:** Summary of laboratory findings at each sepsis episode.

	Episode No.					
	1	2	3	4	5	6
WBC Count (10^6^/L)	80	40	30	1720	1840	9150
Hemoglobin (g/dL)	7.9	9.2	8.2	9.2	8.7	12
Platelet count (10^9^/L)	35	17	26	27	55	45
ANC (10^6^/L)	40	0	0	880	1180	8690
hs-CRP (mg/dL)	4.97	4.86	15.15	4.22	0.12	6.25
AST (mg/dL)	130	32	21	53	53	60
ALT (mg/dL)	352	139	65	145	49	178
Total Bilirubin (mg/dL)	1.21	2.18	3.07	1.85	0.85	1.64
Urea Nitrogen (mg/dL)	33.6	18.7	11.4	3.2	7.1	15.9
Creatinine (mg/dL)	0.57	0.47	0.32	0.33	0.37	0.69

ALT, alanine aminotransferase; ANC, absolute neutrophil count; AST, aspartate aminotransferase; CRP, C-reactive protein.

## Data Availability

Not applicable.
